# Reproductive toxicity and gender differences induced by cadmium telluride quantum dots in an invertebrate model organism

**DOI:** 10.1038/srep34182

**Published:** 2016-09-27

**Authors:** Si-Qi Yan, Rui Xing, Yan-Feng Zhou, Kai-Le Li, Yuan-Yuan Su, Jian-Feng Qiu, Yun-Hu Zhang, Ke-Qin Zhang, Yao He, Xiao-Ping Lu, Shi-Qing Xu

**Affiliations:** 1 School of Biology and Basic Medical Sciences, Medical College, Soochow University, Suzhou 215123, China; 2 National Engineering Laboratory for Modern Silk (NESER), Soochow University, Suzhou 215123, China; 3 Institute of Functional Nano & Soft Materials (FUNSOM), Soochow University, Suzhou 215123, China; 4 Research Center of Cooperative Innovation for Functional Organic/Polymer Material Micro/Nanofabrication, Soochow University, Suzhou 215123, China

## Abstract

Sexual glands are key sites affected by nanotoxicity, but there is no sensitive assay for measuring reproductive toxicity in animals. The aim of this study was to investigate the toxic effects of cadmium telluride quantum dots (CdTe-QDs) on gonads in a model organism, *Bombyx mori*. After dorsal vein injection of 0.32 nmol of CdTe-QDs per individual, the QDs passed through the outer membranes of gonads via the generation of ROS in the membranes of spermatocysts and ovarioles, as well as internal germ cells, thereby inducing early germ cell death or malformations via complex mechanisms related to apoptosis and autophagy through mitochondrial and lysosomal pathways. Histological observations of the gonads and quantitative analyses of germ cell development showed that the reproductive toxicity was characterized by obvious male sensitivity. Exposure to QDs in the early stage of males had severe adverse effects on the quantity and quality of sperm, which was the main reason for the occurrence of unfertilized eggs. Ala- or Gly-conjugated QDs could reduce the nanotoxicity of CdTe-QDs during germ cell development and fertilization of their offspring. The results demonstrate that males are preferable models for evaluating the reproductive toxicity of QDs in combined *in vivo/in vitro* investigations.

Inorganic quantum dots (QDs) have potential as novel biomedical imaging agents with high sensitivity as well as high spatial and temporal resolution^1^,^2^,^3^,^4^. However, biosafety assessments for QDs remain a controversial issue and they are still a major challenge at present^5^,^6^,^7^. Observations of reproductive toxicity are used to evaluate the ecological impact and chronic toxicity in organisms, which are imperative for the application and development of nanochemicals in basic research^8^,^9^. Various QDs are known to be toxic to cells^10^,^11^,^12^,^13^ and different animal species, such as *Caenorhabditis elegans*^14^, *Bombyx mori*^6^,^15^, *Hydra vulgaris*^16^, zebrafish^17^,^18^,^19^, and mice^20^,^21^,^22^, but the reproductive toxicity of QDs and the mechanism of QD toxicity in animals are unclear, especially in males.

Several reports suggest that the clinical utility of QDs could be limited in pregnant females. Indeed, an *in vivo* animal experiment showed that CdTe/CdS QDs may be transferred from female mice to their fetuses across the placental barrier^23^. *In vitro* experiments have demonstrated that CdSe-core QDs induced apoptosis in incubated mouse blastocysts, as well as inhibiting cell proliferation, retarding early post-implantation blastocyst development, increasing early-stage blastocyst death^23^, and reducing the rates of oocyte maturation and fertilization, whereas the same effects were not obtained with ZnS-coated CdSe QDs^24^. A recent report pointed out that high dosages of nanoscale graphene oxide (25 mg/kg mouse) via the tail vein exhibited normal sex hormone secretion and retained normal reproductive activity^25^. One report published earlier this year said that there was no evidence of combined repeated-dose toxicity of silver nanoparticle in rats^26^. While an experiment suggested that silver nanoparticles -treated mice shows that a significant loss of male and female germ cells^26^. Thus, there is much debate regarding the mechanism behind this difference in reproductive toxicity of QDs in male animals. However, it is not clear whether QDs have reproductive toxicity with impacts on the maturation and differentiation of male germ cells.

*In vivo* studies have shown that QDs are non-specifically absorbed and degraded by organs containing reticuloendothelial tissues, such as the liver, spleen, and lymph nodes in *Rattus norvegicus* after injection with QDs^27^,^28^,^29^; thus, no long-term toxicity occurred^27^,^30^. Silicon QDs do not cause obvious toxicity in mice and monkeys^31^. In addition, CdSe/ZnS or CdTe QDs had no direct embryotoxic or teratogenic effects in Wistar rats, but they had adverse effects on the maternal organism. The observed effects of QDs and their long-term accumulation in the maternal organism may increase the risk of adverse effects on embryonic development^32^. However, QDs have a low capacity for crossing the placental barrier as well as no effects on the survival of fetal liver erythroid cells and erythropoiesis in mice^33^. At present, the reproductive toxicity of QDs is still a controversial issue and the possible mechanism involved remains unknown in mammals.

Hydrobios have also been used to assess the reproductive toxicity of QDs. After continuous exposure to sub-lethal doses of CdTe QDs in the living polyps of *Hydra vulgaris*, the treated population founders exhibited impairments in their budding process. The QDs were diluted in the progeny from the second generation onward^16^. After continuous exposure to 0.05–31.25 mg L^–1^ of low level MPA-CdSe or 1–400 nM CdTe QDs coated with thioglycolic acid, zebrafish embryos exhibited malformations and a lower hatch rate^17^,^18^. Moreover, MPA-CdSe induced cellular apoptosis in the head and tail regions of embryos^18^. Pollution with 500 μg/L CdSe QDs led to greater mortality, a lower hatch rate, and more malformations in exposed *Danio rerio*^19^. Cadmium was also detected in the eggs laid by *Fundulus heteroclitus* parents that had been fed 10 μg QDs/day. However, exposure to QDs also decreased the number of abnormal eggs and increased the hatchability rate^34^. Thus, there is much debate regarding the mechanism that underlies these differences in the reproductive toxicity of QDs in various animals, so it would be useful to identify a representative animal reproductive system that could be used as a platform for relevant studies.

In this study, we determined differences in the cytotoxic effects of QDs on males and females; in particular, we determined whether QDs decorated with amino acids have effects on the development of gametes. The invertebrate model organism, the silkworm (*Bombyx mori*), was selected to determine the effects of QDs on gonads and their functions in combined *in vivo/in vitro* experiments. The generation and release of *B. mori* sperm from the spermatotheca can be repeated reliably *in vitro*^35^,^36^,^37^. Indeed, this *in vivo/in vitro* experimental platform has been used successfully to study the toxic effects of QDs on hematopoiesis^6^. This system can avoid the limitations caused by tissue barriers in mammals^38^, because the organs cultured in medium are highly similar to those immersed in hemolymph *in vivo*^39^. Our results suggest that *B. mori* testis is a potentially promising animal model for QD biosafety assessments.

## Results

### Transfer of QDs to the gonads

**Figure 1:**
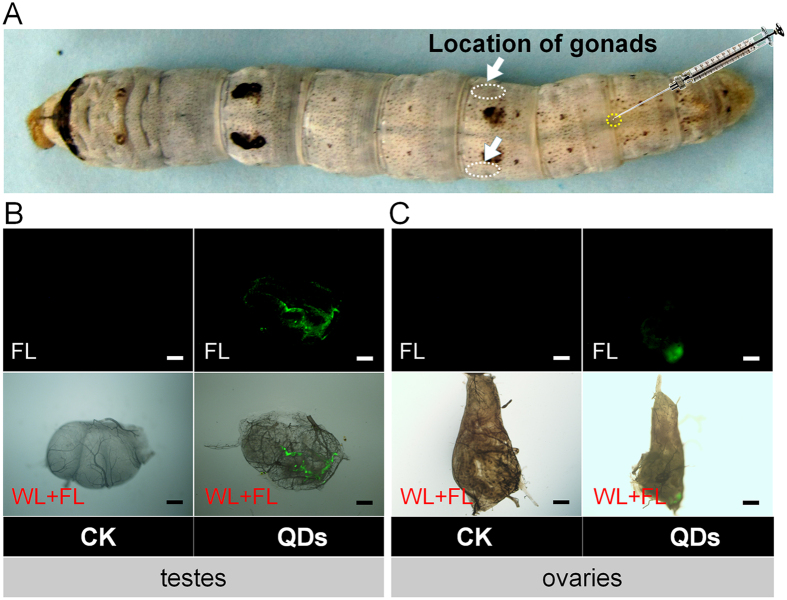
(**A**)Location of the gonads in silkworm larva. QDs were found in the testes (**B**) and ovaries (**C**) by the characteristic green fluorescent. Fifth instar larvae received vascular injection of 0.32 nmol CdTe QDs per larva (10 μL at 32 μM per individual) at 48 h after molting, whereas the control organisms (CK) were injected with the same volume of pure water. The gonads were removed randomly from male larvae at 24 h and female larvae at 48 h after exposure to QDs. WL + FL, merged images showing white light and fluorescent light. FL, fluorescent light. The green fluorescence of QDs was observed at an emission wavelength of 530 nm using a fluorescence microscope (Olympus BX51, Tokyo, Japan). Bar = 100 μm.

In our previous study, we showed that QDs can rapidly enter circulating blood cells in silkworms after dorsal vein injection of 0.08 or 0.32 nmol CdTe QDs per larva (10 μL at 8 μM or 32 μM per individual), thereby reducing the capacity for hematopoiesis^6^. In the present study, a similar dorsal vein injection method was used to expose silkworm hemolymph to 0.32 nmol CdTe QDs per larva (10 μL at 32 μM per individual). The characteristic green fluorescence of QDs was detected in the testes at 24 h after injection (Fig. 1B). Similar results were also obtained in the ovaries at 48 h after injection (Fig. 1B). The transfer of QDs from the hemolymph circulation system to the reproductive system emphasizes the utility of this system for studying potentially reprotoxic effects. We further investigated the effects of modifying the surfaces of QDs with amino acids on their transfer into the reproductive system. They found that the characteristic green fluorescence of QDs was detected in the testes and ovaries at 12 h after exposure to 0.64 nmol CdTe QDs, QDs-Ala or QDs-Gly (10 μL at 64 μM per individual), but the proportion of glands that exhibited weak fluorescence in the QDs-Ala or QDs-Gly treatment groups was higher than that in the QDs group (Fig. S1), thereby implying that Ala- or Gly-capped CdTe QDs may reduce the transfer and accumulation of QDs to the gonads.

### Impact of exposure to QDs on oviposition and fertilization

**Figure 2:**
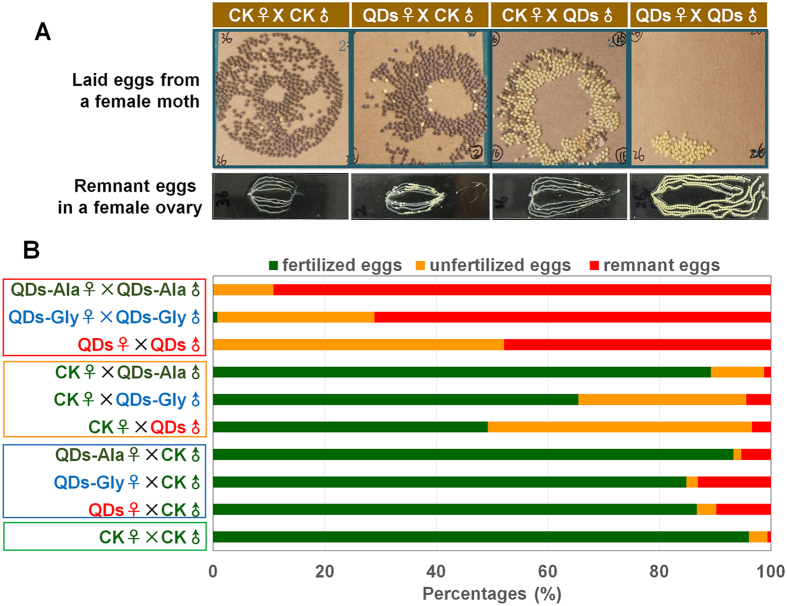
Effects of QDs on oviposition and fertilization. (**A**) Type of egg laid and remaining eggs in the ovaries. (**B**) Egg-laying habits and fertilization of eggs. Fifth instar larvae received vascular injection of 0.32 nmol CdTe QDs per individual (10 μL at 32 μM) at 48 h after molting, whereas the control organisms (CK) were injected with the same volume of pure water. The larvae were reared on fresh mulberry leaves at 25 °C with a photoperiod of 12 h light and 12 h dark until they became adult moths. The female adults were mated for 4 h with descriptive mates and then laid eggs 24 h. Next, the ovaries were removed from the female moths to determine the remaining eggs. The yellow unfertilized eggs and puce fertilized eggs were measured at 72 h after oviposition. n = 5 pairs.

In *B. mori*, the 5th larval stage, which lasts about 1 week, is the key stage for spermatogenesis in the testes and oogenesis in the ovaries^37^. In this study, 5th instar larvae in the early stage of the silkworm life cycle were exposed to 0.32 nmol CdTe QDs via a single dorsal vein injection. The results showed that QDs had a significant impact on oviposition by the adults during the later stage of the life cycle and fertilization of the offspring eggs. When a pair of male and female silkworms was exposed to QDs, almost all of the eggs laid by female moths were not capable of being fertilized and some eggs in their ovaries were not laid, thereby indicating that QDs can severely affect reproductive toxicity (Fig. 2A). It should be noted that if only one of the pair was exposed to QDs, the impact of QDs on oviposition and fertilization varied greatly. Exposing female larvae to QDs changed the oviposition behavior of the female moths, including increased eggs remaining in the body, but the fertilization of the laid eggs was almost normal. When a male larva was exposed to QDs, the male moth mated later with a normal female moth and the fertilization rate of the laid eggs was significantly reduced (Fig. 2A). This phenomenon was confirmed by quantitative comparisons using multiple duplicate individuals (Fig. 2B and Fig. S2).

Silkworm larvae were further exposed to QDs-Ala or QDs-Gly, and the results showed that the adverse effects of these QDs on oviposition and fertilization were lower than those of the QDs alone, where the toxicity of QDs-Ala was the lowest. It should be noted that when both the male and female were exposed to QDs-Ala or QDs-Gly, the rate or number of unfertilized eggs were significantly lower than those in the QDs group, but the number of eggs remaining in the body increased significantly and it was even higher than that in the group where both sexes were exposed to QDs (Fig. 2B and Fig. S2D). This indicates that the amino acid modified-QDs still had severe effects on reproductive toxicity and they also seemed to influence the oviposition behavior of the female moths.

The sex-dependent differences in the reproductive toxicity of QDs were still observed after modifying the QDs with amino acids, where male exposure to QDs significantly reduced the rate of fertilized eggs, whereas female exposure only slightly reduced the oviposition rate (Fig. 2B). As shown in Fig. S2A, female exposure to different QDs did not lead to obvious declines in the total number of eggs. These results suggest that the three QDs used in the experiments did not severely affect the development of female reproductive cells; therefore, QDs might have serious adverse effects on the development of male reproductive cells.

### Gonadal morphology after exposure to QDs

**Figure 3:**
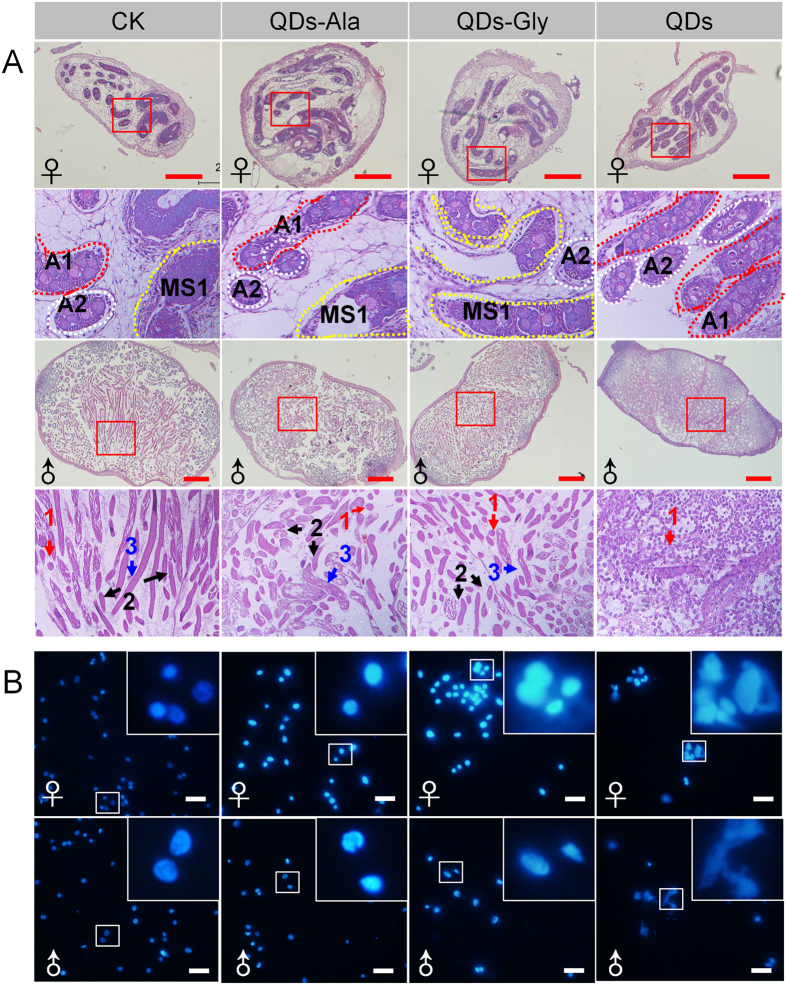
Impact of QDs on the development of silkworm gonads and the nuclear morphometry of gametes. (**A**) The HE staining of testes or ovaries. A1 and A2 indicate the early and late anagen stages in the oogonia, respectively. During this stage, an oogonium differentiated into one primary oocyte and seven nurse cells. MS1 indicates the early stage of maturity; 1–3 indicate the stage of primary spermatocytes in spermatocysts, the spermatocysts during spermiogenesis, and the stage of spermatodesms, respectively. (**B**) The DAPI staining of histiocytes in testes or ovaries. Fifth instar larvae received vascular injection of 0.32 nmol CdTe QDs per individual (10 μL at 32 μM) at 48 h after molting, whereas the control organisms (CK) were injected with the same volume of pure water. The gonads were removed from older larvae at 120 h after exposure to QDs and used for HE or DAPI staining. For DAPI staining, the histiocytes were stained with DAPI after thoroughly splitting from the outer membrane of testes and ovaries with forceps.♀ indicates female and ♂ indicates male. Bar = 100 μm in (**A**) and 10 μm in (**B**).

At 120 h after the 5th instar larvae were exposed to QDs, hematoxylin and eosin (HE) staining of the gonad slices (Fig. 3A) showed that the germ cells in the testes and ovaries developed at a significantly slower rate than those in the control water-treatment group. In the ovaries of the QDs treatment group, only oogonia in the anagen phase were present and there were no signs of mature eggs at the early stage, while only primary spermatocytes were observed in spermatocysts in the testes, and spermatocysts undergoing spermiogenesis and the spermatodesms that were found in large numbers in the control group were not observed, thereby indicating that exposure to QDs reduced the development rate of germ cells at the early and middle stages in the gonads.

The development rate of the germ cells in female ovaries at 120 h after exposure to QDs-Ala or QDs-Gly was faster than that in the QDs group, and there was almost no difference from the control group (Fig. 3A), which further demonstrated that female exposure to QDs-Ala or QDs-Gly caused no significant differences in the total number of eggs (Fig. S2B) as well as the number of fertilized eggs in a single moth (Fig. S2C) in the treatment and control groups. However, although the germ cells in male testes developed at a faster rate at 120 h after QDs-Ala or QDs-Gly exposure than those in the QDs treatment group, the rate was still slower than that in the control group (Fig. 3A), thereby confirming that the reproductive toxicity of QDs mainly affected male fertility.

Nuclear division and damage to hemocytes were detected using 4′,6-diamidino-2-phenylindole (DAPI) to stain the cell nucleus. The results showed that some nuclei were damaged in the germ cells of both the ovaries and testes at 120 h after exposure to QDs, which was evident as deformation or shrinkage of the nuclei, but the damaged nuclei were more obvious in the testes. There was little difference in the nuclear morphology in the groups exposed to QDs-Ala or QDs-Gly and the control group, and there were no significant differences between sexes (Fig. 3B).

### Quantitative analysis of the impact of exposure to QDs on male germ cell development

**Figure 4:**
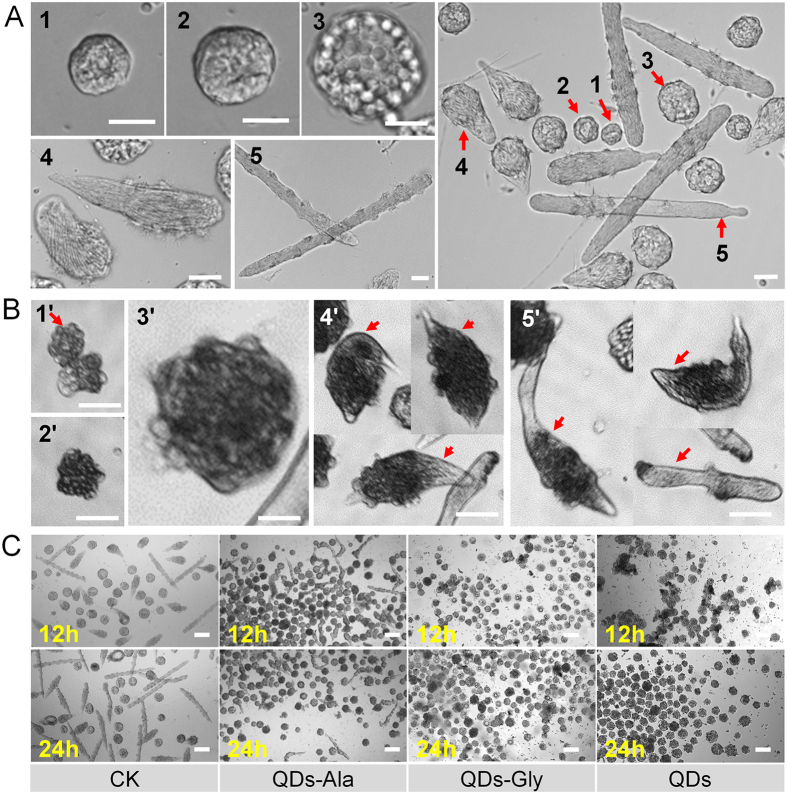
Effects of QDs on spermateleosis in *Bombyx mori*. (**A**) Natural morphology of spermatocysts in the different stages. 1–3 indicate the stages with 64, 128, and 256 primary spermatocytes in spermatocysts, respectively; 4 indicates the spermatocysts during spermiogenesis; 5 indicates the stage of spermatodesms. **(B**) The culture of male spermatocysts after *in vivo* exposure to QDs. Cacoplastic morphology of spermatocysts characterized by warping, apoptosis, necrosis, or damaged membranes. 1′–3′ indicate the stages with 64, 128, and 256 cacoplastic primary spermatocytes in spermatocysts, respectively; 4′ indicates the cacoplastic spermatocysts during spermiogenesis process; 5′ indicates the stage of cacoplastic spermatodesms. **(C)** Spermateleosis was affected by the duration of exposure *in vivo*. The duration of exposure *in vivo* was the time until the removal of the testes after exposure to QDs. Fifth instar larvae received vascular injection of 0.32 nmol CdTe QDs, QDs-Ala, or QDs-Gly per larva (10 μL at 32 μM per individual) at 48 h after molting, whereas the control organisms (CK) were injected with the same volume of pure water. Spermatocysts were removed from male larva at 12 h or 24 h after exposure to QDs, and then cultured for 168 h *in vitro* without QDs. Bars = 100 μm in (**A**,**B**), and 200 μm in (**C**).

There have been no previous quantitative assessments of the impact of exposure to QDs on animal male germ cell development and a suitable research platform is not available. In order to quantitatively assess the impact of exposure to QDs on the development of silkworm male germ cells, we established an *in vitro* method for the culture of male spermatocysts after *in vivo* exposure to QDs in a combined *in vivo/in vitro* approach. As shown in Fig. 4A, the *in vitro*-cultured normal spermatocysts could complete the whole *in vivo* spermatogenesis process from spermatogonia to mature sperms, including primary spermatocytes, spermatocysts during the spermiogenesis process, and the spermatodesms stage.

The testes were removed at 24 h after exposure to QDs and although they left the environment containing QDs, the growth and development of the spermatocysts were affected significantly after *in vitro* cultivation for 168 h. Figure 4B shows the typical poorly-developed spermatocysts during the development from spermatogonia to mature sperms, which includes primary spermatocytes, spermatocysts during spermiogenesis, and the spermatodesms stage.

Figure 4C shows the morphology of the spermatocysts after *in vitro* cultivation for 168 h where the testes were removed at 12 h or 24 h after exposure to QDs. After *in vitro* cultivation for 168 h, for the spermatocysts in the control group where the larvae were not exposed to QDs, mature spermatodesms formed and poorly-developed spermatocysts appeared only rarely, regardless of whether the testes were removed at 12 h or 24 h. By contrast, in the groups exposed to QDs, no mature spermatodesms were observed and most of the spermatocysts did not develop or they developed poorly, while atrophied and necrotic spermatocysts even appeared. In the group where the testes were removed at 12 h after exposure, most of the spermatocysts were atrophied and necrotic, thereby indicating that previous *in vivo* exposure to QDs had severe adverse effects on the later development of spermatocysts even after they left the environment containing QDs. Exposure to QDs-Ala or QDs-Gly also had severe adverse effects on the development of spermatocysts, but their toxicity was lower than that of the QDs alone. After *in vitro* cultivation, there were fewer atrophied and necrotic spermatocysts in the QDs-Gly exposure group compared with the QDs exposure group. The adverse effects of exposure to QDs-Ala were lower, where the numbers of atrophied and necrotic spermatocysts were lower, and mature spermatodesms also appeared after *in vitro* culture for 168 h. These results indicate that modification with amino acids reduced the toxic effects of QDs on the development of spermatocysts, but the effects of different amino acid modifications differed.

Figure 4C also shows that the time when the testes were removed from the *in vivo* QD-exposure environment and the *in vitro* cultivation of spermatocysts started had different effects on the degree of spermatocyst development, which suggests that the potential toxicity of QDs continued to accumulate in the target tissues (i.e., the gonads) of the animals after exposure to QDs, whereas detoxification occurred in the animals. To verify this hypothesis, we quantitatively investigated the impact of the *in vitro* cultivation of spermatocysts in a QD-free environment from which the testes were taken.

**Figure 5:**
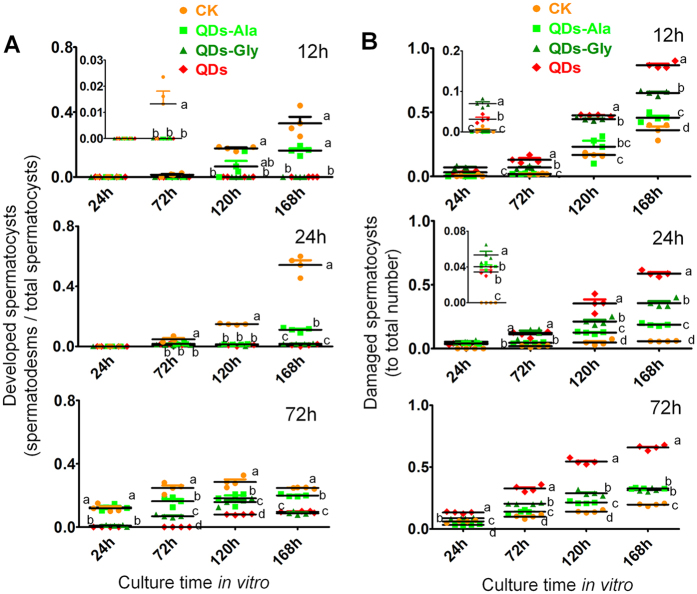
Spermateleosis was affected by the duration of exposure to QDs *in vivo* and the duration of culture free from QDs *in vitro*. (**A**) Ratio of spermatodesms relative to the total number of spermatocysts. (**B**) Ratio of damaged spermatocysts relative to the total number of spermatocysts. Fifth instar larvae received vascular injection of 0.32 nmol CdTe QDs, QDs-Ala, or QDs-Gly per larva (10 μL at 32 μM per individual) at 48 h after molting, whereas the control organisms (CK) were injected with the same volume of pure water. Spermatocysts were removed from male larvae at 12 h, 24 h, or 72 h after exposure to QDs and then cultured *in vitro* without QDs. The duration of exposure *in vivo* was the time until the removal of testes after exposure to QDs. Samples marked with the same letter do not differ significantly from each other, *P < *0.05 (n = 3 microscopic fields of view).

Figure 5A shows the changes in the ratio of spermatodesms relative to the total number of spermatocysts when the testes were removed at 12 h, 24 h, or 72 h after exposure to QDs and then cultured *in vitro*. In the control group, the ratio increased significantly with the time of *in vitro* culture, where the ratio of spermatodesms relative to the total number of spermatocysts was 1.64 times higher at 168 h when the testes were removed at 24 h and the spermatocysts were cultivated *in vitro* compared with that when the testes were removed at 12 h (Fig. 5A). The ratio of damaged spermatocysts was also 58% lower when the testes were removed at 24 h compared with that when they were removed at 12 h (Fig. 5B).

It should be noted that when the testes were removed at 72 h, many spermatodesms were generated in the early stage of cultivation (e.g., 24 h) in the control group, but the spermatodesms increased little in the late stage, where the ratio of spermatodesms relative to the total number of spermatocysts was even lower after 168 h of cultivation compared with that after 120 h of cultivation (Fig. 5A). Further investigations showed that there were more damaged spermatocysts when the testes were removed at 72 h compared with that when they were removed at 24 h, and the damaged spermatocysts appeared significantly earlier during the cultivation process compared with that when they were removed at 12 h (Fig. 5B), thereby indicating that 72 h was not the best time for removing the testes for the *in vitro* cultivation of spermatocysts. It is possible that the spermatocysts had begun to differentiate into spermatodesms in the testes by this time, which made them vulnerable to injury during the operation. According to the results of the *in vitro* cultivation of spermatocysts, the frequency of damaged spermatocysts was the lowest when the testes were removed at 24 h after the injection of water in the control group, and the spermatocysts developed into spermatodesms in a similar manner to that *in vivo*.

When the testes were removed at 12 h or 24 h after exposure to QDs for *in vitro* cultivation of spermatocysts, the spermatocysts rarely developed into spermatodesms. When the testes were removed at 72 h after exposure, a small number of spermatodesms appeared in the later stage after cultivation for 120 h, but the spermatodesms appeared significantly later compared with the control and the ratio of spermatodesms relative to the total number of spermatocysts was significantly lower than that in the control (Fig. 5A). The ratio of damaged spermatocysts was also significantly higher than that in the control group (Fig. 5B). These results indicate that the QDs had obvious toxic effects on male reproduction. It should be noted that when the testes were removed at 72 h after exposure to QDs and the spermatocysts were cultured for 168 h, the ratio of damaged spermatocysts was lower than that when the testes were removed at 12 h after exposure, and not significantly different from that when the testes were removed at 24 h after exposure. As the time when the testes left the QDs environment increased, the ratio of damaged spermatocysts changed in a U-shaped pattern from 12 h to 72 h after exposure to QDs (Fig. 5B). These results indicate that as the time after exposure increased, the male gonads potentially accumulated toxic QDs but the animals also reduced the toxicity of the QDs via detoxification, where the latter appeared to have a greater effect.

The ratio of spermatodesms relative to the total number of spermatocysts was significantly higher in the Ala- or Gly-modified QDs exposure groups compared with that in the QDs exposure group, where the ratio was even higher in the QDs-Ala exposure group (Fig. 5A). The ratio of damaged spermatocysts was significantly lower in the amino acid-modified groups than the QDs exposure group, but the ratio was lowest in the QDs-Ala exposure group (Fig. 5B). These results indicate that linking amino acids to QDs significantly reduced their reproductive toxicity and the adverse effects of QDs-Ala exposure were even lower. It should be noted that similar to the results obtained after exposure to the QDs, after *in vitro* cultivation for 168 h, the differences in the ratio of spermatodesms relative to the total number of spermatocysts between the Ala- or Gly-modified QDs exposure groups and the control group were greater when the testes were removed at 24 h compared with that when removed at 12 h, while the differences when removed at 72 h were smaller than those when removed at 24 h (Fig. 5A). These results indicate that within 24 h of exposure, the male gonads accumulated toxic QDs, but the effects of QD detoxification increased rapidly in the male gonads up to 72 h, or the repair effect may have increased rapidly in the male gonads.

### ROS levels in the gonads

**Figure 6:**
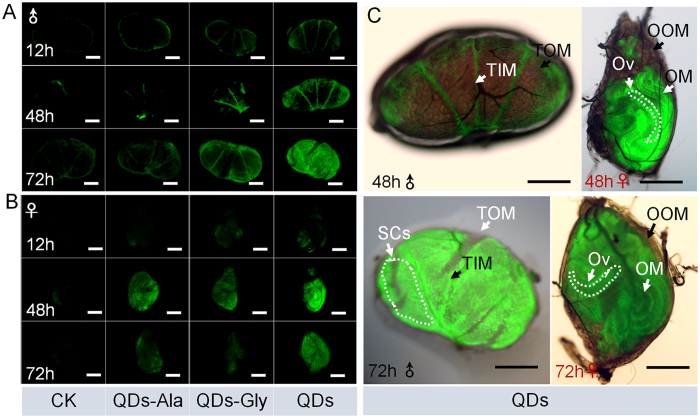
Effects of the duration of exposure to CdTe QDs on the levels of reactive oxygen species (ROS) in testes (**A**) and ovaries (**B**). (**C**) Merged images showing the organizational orientation of ROS in gonads. Fifth instar larvae received vascular injection of 0.32 nmol CdTe QDs, QDs-Ala, or QDs-Gly per larva (10 μL at 32 μM per individual) at 48 h after molting, whereas the control organisms (CK) were injected with the same volume of pure water. ROS were stained at 12 h, 48 h, or 72 h after exposure. TOM, testis outer membrane. TIM, testis inner membrane. SCs, spermary cells. OOM, ovarian outer membrane. OM, ovariole membrane. Ov, ovarioles. Bars = 200 μm.

Figure 6 shows that at 12 h after exposure to QDs, the ROS levels in the testes were higher compared with the control group and they continued to increase until 72 h after exposure, while the ROS levels in the ovaries were also higher than those in the control group at 12 h to 72 h after exposure to QDs (Fig. 6A). In contrast to the testes, although the ROS levels increased in the ovaries at 12–48 h after exposure, the levels were lower at 72 h after exposure compared with those at 48 h after exposure (Fig. 6B). The merged images in Fig. 6C show the organizational orientation of ROS in the gonads after exposure to QDs. In the testes, when the ROS levels were low, ROS mainly occurred in the inner membranes of the testis, which comprise squamous cells. As the ROS levels increased, they gradually appeared in the spermary cells. Very low levels of ROS were detected in the outer membrane of the testis, which contains no cells. In the ovaries, ROS also occurred rarely in the ovarian outer membrane, but they appeared very rapidly in the ovariole membrane, and even in the ovarioles. These results indicate that exposure to QDs induced the generation of ROS in the internal gonadal tissues and even in the germ cells. The changes in the ROS levels varied in the male and female gonads, but the ROS levels induced by exposure to QDs decreased faster in the ovaries compared with the testes, thereby indicating that the ovaries have a stronger oxidative stress regulatory function than the testes.

Figure 6A,B also shows that the locations of ROS in the gonads of the Ala- or Gly- QDs exposure groups were similar to those in the QDs exposure group. After exposure to QDs-Ala or QDs-Gly, the ROS levels in the gonads were higher than those in the control group, but significantly lower than those in the QDs exposure group, and the ROS levels decreased in the ovaries at 72 h after exposure to levels similar to those in the control group. These results indicate that linking amino acids to CdTe QDs could decease the generation of ROS in gonadal cells, which may explain why the reproductive toxicity of QDs-Ala or QDs-Gly was lower than that of QDs.

### Degree of mitochondrial damage in gonadal cells and the transcription levels of an apoptosis signaling gene

The results in above Fig. 3 obtained after DAPI staining also showed that the apoptotic cell nucleus in testes or ovaries was observed after exposure to QDs. Figure 6 results shown that QDs induced the generation of ROS in gonadal cells. In order to explore the relationship between ROS generation and nuclear damage in gonadal cells, we investigated the effects of exposure to QDs on the transcription levels of an apoptosis signaling gene, as well as the structural damage to mitochondria, which was the molecular target of apoptotic signal transduction.

**Figure 7:**
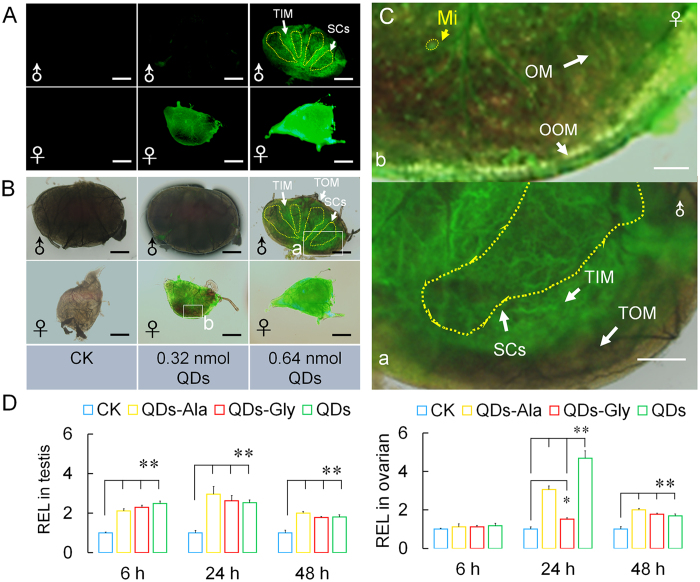
Mitochondrial staining in the gonads and the *BmDronc* gene expression levels under after exposure to CdTe QDs. Fifth instar larvae received vascular injection of CdTe QDs, QDs-Ala, or QDs-Gly per larva (10 μL at 32 μM per individual in Fig. 8A–F, and 10 μL at 64 μM in Fig. 8A–C per individual) at 48 h after molting, whereas the control organisms (CK) were injected with the same volume of pure water. **(A**) Fluorescence images showing the degree of mitochondrial damage in testes (♂) and ovaries (♀). (**B**) Merged images showing the organizational orientation of damaged mitochondria in the gonads. (**C**) Enlarged images showing the organizational orientation of damaged mitochondria in the gonads. a and b indicate enlarged images of the testes and ovaries within the box in Fig. 8A, respectively. b indicates an enlarged image of the ovary within the box in Fig. 8B. The gonads were collected and stained at 24 h after exposure to QDs. Mi, mitochondria. TOM, testis outer membrane. TIM, testis inner membrane. SCs, spermary cells. OOM, ovarian outer membrane. OM, ovariole membrane. Ov, ovarioles. Bars = 200 μm in (**A**,**B**), and 50 μm in (**C**). **(D**) Relative expression level (REL) of the *BmDronc* gene in gonads. The testes and ovaries were collected for qRT-PCR at 6 h, 24 h, and 48 h after exposure. The *BmDronc* gene transcript level was analyzed by qRT-PCR. The BmRP49 gene was selected as an internal control. *P < 0.05 and **P < 0.01 indicate significant differences (n = 3 technical repeats).

The staining of mitochondria in the gonadal cells showed that mitochondrial debris, which was dyed green, was present in female gonadal cells at 24 h after the injection of 0.32 nmol CdTe QDs via the dorsal vein (Fig. 7A,B). Star-shaped swollen mitochondria were visible due to changes in membrane permeability, which are typical during apoptosis (Fig. 7C). When the concentration of QDs increased to 0.64 nmol, green mitochondrial debris was also found in the testis cells, where the green fluorescence in the testis inner membrane cells, particularly the four sperm chambers, was significantly greater than that in the germ cells inside the sperm chambers. In the female ovaries, the CdTe-QDs appeared to associate with the isolated mitochondria according to their inherent fluorescence (Fig. 7A,B).

As shown by the enlarged image in Fig. 7C, the mitochondrial debris with green fluorescence in the sperm chambers was mainly present in the membranes of spermatocysts, the morphology of which varied from circular shapes to strips and other linear shapes. When the concentration of QDs increased to 0.64 nmol, the green fluorescence from mitochondrial debris in the ovarian cells was brighter than that with QDs at 0.32 nmol, and the shape of the fluorescence changed from star shapes at a low dose to larger objects, but the fluorescence intensity was still greater at the periphery of the ovaries than the central region (Fig. 7A,B). These results indicate that the mitochondria in the gonadal cells were damaged after exposure to QDs for 24 h, and the extent of this damage was greater in the female gonads than the male gonads.

In addition, the green fluorescence in Fig. 7 shows the changes in the locations of the mitochondrial debris inside gonadal cells, which were very similar to the changes in the locations of ROS, as shown in Fig. 6, where the locations spread from the inner membrane to the germ cells in the spermatocysts or ovarioles over time. These results suggest that the mitochondrial damage in gonadal cells caused by exposure to QDs might be induced by the generation of ROS.

We also investigated changes in the transcription levels of BmDronc, which is a gene related to the apoptosis signaling pathway. Figure 7D shows that after dorsal vein injection of 0.32 nmol CdTe QDs, the *BmDronc* gene transcript levels were significantly upregulated in the testis and ovarian cells. The transcription levels of the *BmDronc* gene were already significantly upregulated in testis cells at 6 h after exposure, which was earlier than the change in ovarian cells, thereby suggesting that QDs induced enhanced apoptotic signaling in the testis cells earlier than the ovarian cells.

Figure 7D also shows that there were no significant differences in the transcription levels of the *BmDronc* gene in testis cells from the Gly or Ala- conjugated CdTe QDs groups and the QDs group, but at 24 h after exposure, the *BmDronc* gene transcription level was lower in ovarian cells from the QDs-Gly group than the QDs group, which suggests that the reduced toxicity of the amino acid-conjugated CdTe QDs might not be mediated mainly via the apoptosis pathway.

### Lysosomes and the transcription levels of autophagy signaling genes in gonad cells

Lysosomes are very sensitive to oxidative stress, where mild oxidative stress in cells can make the lysosomal membrane unstable and the increased membrane permeability induces autophagy^40^,^41^,^42^. Therefore, we investigated changes in the transcription levels of autophagy signaling-related genes in gonad cells as well as the production of intracellular lysosomes.

**Figure 8:**
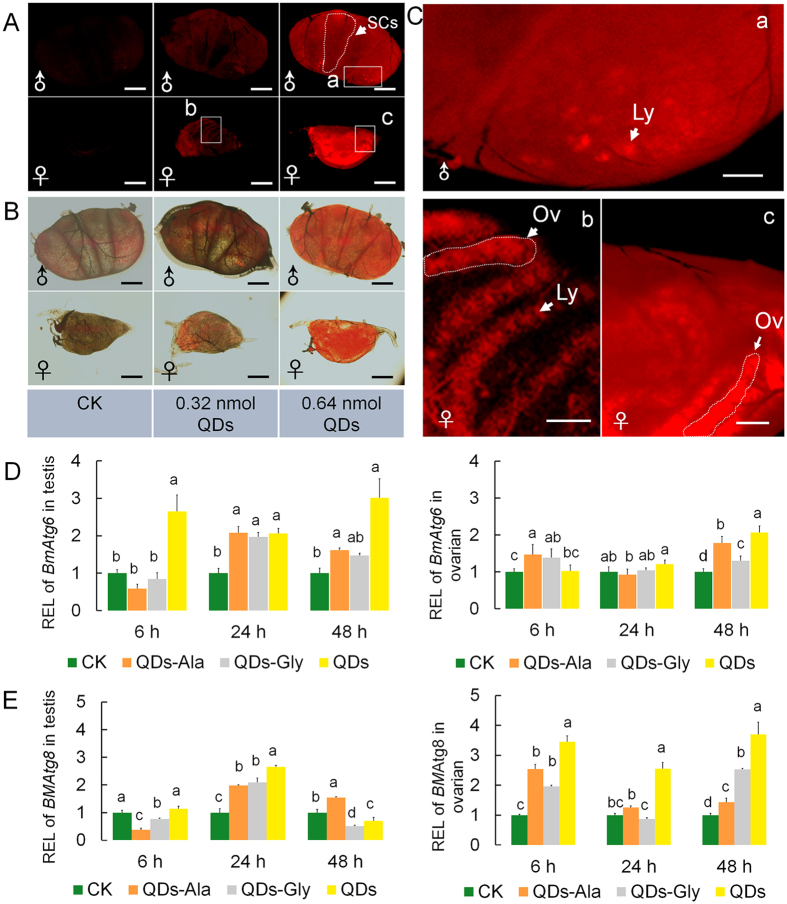
Staining of lysosomes in gonads and the *BmAgt6*/*BmAtg8* gene expression levels after exposure to CdTe QDs. Fifth instar larvae received vascular injection of CdTe QDs, QDs-Ala, or QDs-Gly per larva (10 μL at 32 μM per individual in Fig. 8A–F, and 10 μL 64 μM in Fig. 8A–C per individual) at 48 h after molting, whereas the control organisms (CK) were injected with the same volume of pure water. **(A**) Fluorescence images showing the lysosome level in testes (♂) and ovaries (♀). (**B**) Merged images showing the organizational orientation of dyed lysosomes in gonads. (**C**) Enlarged images showing the organizational orientation of dyed lysosomes in gonads. a and c indicate the enlarged images of testes and ovaries within the box in Fig. 8A, respectively. b indicates the enlarged image of ovaries within the box in Fig. 8A. The gonads were collected and stained at 24 h after exposure to QDs. Ly, lysosome. SCs, spermary cells. Ov, ovarioles. Bars = 200 μm in (**A**) and (**B**), and 50 μm in (**C**). Relative expression levels (REL) of the *BmAtg6* and *BmAtg8* genes in testes (**D**) and ovaries (**E**) determined by qPCR. The gonads were collected for qRT-PCR at 6 h, 24 h, and 48 h after exposure to QDs. The *BmAtg6* and *BmAtg8* gene transcript levels were analyzed by qRT-PCR. The *BmRP49* gene was selected as an internal control. Samples marked with the same letter do not differ significantly from each other, *P < *0.05 (n = 3 technical repeats).

The lysosomal staining results shown in Fig. 8A and B demonstrates that lysosomal-specific red fluorescence was present in the testis and ovarian cells at 24 h after exposure to 0.32 nmol CdTe QDs. When the concentration of QDs increased to 0.64 nmol, the red fluorescence in the testes and ovaries indicated that the abundance of lysosomes increased. In contrast to the mitochondrial staining results shown in Fig. 7, the increased levels of lysosomes in gonad cells caused by exposure to QDs did not exhibit obvious gender differences, thereby suggesting that after exposure to the low dose of QDs employed in the experiment, oxidative stress could induce the formation of lysosomes in both male and female gonadal cells. The enlarged image in Fig. 8C shows that the mitochondria generating positions were different from those in Fig. 7, where the red fluorescence from lysosomes occurred mainly in germ cells inside the gonadal tissues, with bright red scattered spermatocysts with various shapes visible through the outer membrane of the testes (Fig. 8C-a), and the red fluorescence in female ovarioles was arranged in the shape of spinal bone spurs or combined into a single piece when more lysosomes were generated (Fig. 8C-b,c).

We investigated the transcription levels of *BmAtg6* and *BmAtg8* genes, which are associated with the regulation of autophagy. Figure 8D shows that after injecting 0.32 nmol CdTe QDs, the transcription levels of the *BmAtg6* and *BmAtg8* genes tended to be upregulated in the testis and ovarian cells, but the time when upregulation of these two genes occurred in the testes and ovaries differed in the period from 6–48 h after exposure. The *BmAtg6* gene transcription levels in testis cells were always significantly higher than those in the control group during 6–48 h after exposure to QDs, while the transcription levels in ovarian cells were higher than those in the control group until 48 h after exposure to QDs. The *BmAtg8* gene transcription levels in ovarian cells were always significantly higher than those in the control group during 6–48 h after exposure to QDs, but the transcription levels in testis cells were only higher than those in the control group at 24 h after exposure, and they were even lower than those in the control group at 48 h after exposure. Gly- or Ala-conjugated CdTe QDs also affected the *BmAtg6* and *BmAtg8* gene transcription levels in the testis and ovarian cells. The *BmAtg6* gene transcription levels in ovarian cells at 6 h after exposure to QDs-Ala and the *BmAtg8* gene transcription levels in testis cells at 48 h after exposure to QDs-Ala were higher than those in the group exposed to QDs for the same time, but the overall transcription levels in the Gly- or Ala-conjugated CdTe QDs exposure groups were downregulated compared with the QDs exposure group (Fig. 8D), which suggests that the reduced toxicity of amino acid-conjugated CdTe QDs might be related to downregulation of the transcription levels of autophagy regulation signaling genes.

## Discussion

### Reproductive toxicity of QDs exhibited gender differences

It has been reported that QDs have severe adverse effects on fertilization in animals and the hatching of oviparous animals. For example, exposure to CdSe-core QDs in mice^24^ and *Danio rerio*^19^ decreased their egg fertilization rates, while *Hydra vulgaris* exhibited impaired budding with CdTe QDs^16^. QDs also decreased the hatching rate in *Danio rerio*^17^,^18^. However, it is not clear whether QDs have reproductive toxicity with impacts on the maturation and differentiation of male germ cells.

In our previous study, the tracking analysis of CdTe-QDs or amino acid-modified QDs in multiple tissues of silkworm were performed^15^. And the dynamic distribution of QDs in testes or ovaries are supposedly-written-by the findings. The present study demonstrated that the characteristic fluorescence of CdTe QDs appeared earlier in silkworm testes and the fluorescence intensity, which represents the amount of accumulated QDs, was also stronger than that in the ovaries (Fig. 1 and Fig. S1), thereby indicating that gender differences might exist in the speed and efficiency with which QDs enter the gonads. The reason may be related to the difference of size and the structure between testis and ovary. Or, at least, the size of ovary is times bigger than this of testes. The results of further mating experiments also showed that although CdTe QDs only had slight effects on egg production and the number of eggs laid by females, the severe impact of CdTe QDs on the fertilization rate was due to their effects on males (Fig. 2 and Fig. S2). Our observations of gonadal development (Fig. 3) and a combined quantitative *in vivo/in vitro* investigation of the cultivation of spermatocysts also demonstrated that exposure to QDs was closely related to poor development of the male germ cells (Figs 4 and 5). These results indicate that exposure to QDs might have severe adverse effects on the quantity and quality of sperm formation in male silkworms, and thus we suggest that studies of the reproductive toxicity of QDs should focus on observation in males.

Previous studies have shown that the biological toxicity of QDs is affected greatly by their coating materials^11^. Recently, it was reported that linking a polypeptide homologous to human protein hCD47 with QDs reduced the clearance rate of QDs by phagocytes in mice^43^. Different QDs, such as CdSe-Cys, CdTe-Cys, and ZnS-Cys, or different surface coverings, such as CdSe-MPA (mercaptopropionic acid) and CdSe-Cys, have significantly different toxic effects in human breast cancer MCF-7 cells, where the extent of oxidative stress in cells also varies^44^. The toxicity of CdSe-SiO_2_ is lower than that of CdSe-MAA (methacrylic acid) or CdSe-MPA in COS-7 cell lines, NIH 3T3 cell lines, and human hepatoma cell lines^45^. Surface covered PEI (Polyetherimide) can reduce the toxic effects of CdSe QDs during the passage of human cervical carcinoma cell lines^46^. Mercaptosuccinic acid-capped Cd QDs^47^, CdSe/ZnS core-shell QDs, or CdTe QDs stabilized by 3-mercaptopropionic acid^14^ and cadmium have different effects on the development of *C. elegans*, and their mechanisms are even different. In the present study, our combined *in vivo/in vitro* investigation of the cultivation of spermatocysts demonstrated that Ala- or Gly-conjugated QDs significantly reduced the toxic effects on sexual glands and reduced the amount of ROS generated in the tissues of *B. mori*. It is known that the toxic effects of QDs differ from those of their bulk counterparts. Thus, nanoparticles with the same chemical composition differ in their toxicological properties, where the differences in toxicity depend upon their size, shape, and surface covering. Many *in vitro* and *in vivo* experiments have demonstrated that surface modifications influence the toxic effects of QDs on cellular proliferation^48^. These results were also confirmed in hemocytes and hematopoietic organs based on our research into hematopoiesis toxicity^15^. The Ala- or Gly-conjugated QDs effectively reduced the amount of reactive oxygen species (ROS) induced by QDs in animal tissues and significantly reduced the toxic effects of CdTe QDs on blood cells and hematopoiesis^15^. The experiments performed in the present study also demonstrated that Ala- or Gly-conjugated QDs reduced the apoptosis of nuclei in gonadal cells induced by CdTe QDs, and the effects of nanotoxicity on the development of gonadal cells were mitigated (Fig. 3). These results suggest that studies of the reproductive toxicity of QDs should focus on the surface modification of QDs.

### Possible mechanisms of reproductive toxicity caused by QDs

Many *in vitro* experiments have demonstrated that QDs aggregate in intracellular particles and lysosomes after entering cells^10^, thereby inducing toxicity by causing changes in the morphology and structure of mitochondria, as well as impairing their functions and stimulating biogenesis^49^, where their degradation releases heavy metal ions^50^ and cell death occurs^11^,^51^. QDs may also damage intracellular proteins, lipids, and DNA by generating ROS^13^, which can have synergistic effects to elicit cytotoxicity^52^.

Some studies have shown that CdTe QDs can bind to type I transmembrane glycoprotein FAS receptors on cell membranes, recruit caspase-8 to obtain a high local concentration and activate FAS, and further activate caspase-1,3,7 and other members, thereby promoting apoptosis in the cells where FAS proteins are located^53^,^54^. The entry of QDs into cells through the cell membrane results in mitochondrial swelling, collapse of the membrane potential, and decreased respiration, as well as promoting cells to produce more cytochrome C, initiate the caspase-8 pathway, and elicit apoptosis^45^,^49^,^55^,^56^. In addition, the cascade caused by ROS can induce the generation and rupture of lysosomes, where hydrolytic enzymes are released into the cytoplasm to rapidly initiate programmed cell death^10^.

In the present study, large amounts of ROS were produced in the gonads of silkworms after exposure to CdTe QDs. Very low amounts of ROS were present in the outer membranes of the gonads and they were found mainly in the membranes of testis spermary cells and spermatocysts, while significantly more ROS were detected in the membranes of ovarioles in the ovaries compared with other locations. ROS fluorescence was also detected in the germ cells of gonads (Fig. 6). These results suggest that the possible mechanism responsible for the reproductive toxicity of CdTe QDs was similar to the toxicity of QDs in cultured cells, which was mediated via the effects of ROS. The staining results obtained for mitochondria (Fig. 7) and lysosomes (Fig. 8) also showed that the mitochondrial debris and lysosomal levels also increased in the locations where ROS were detected inside the gonads, where a dose-dependent effect was observed. DAPI staining of the gonadal cells confirmed that the QDs caused nuclear damage in the testes and ovaries (Fig. 3). Further investigations of the transcription levels of genes associated with autophagy and regulation of the apoptosis signaling pathway also showed that the vascular injection of QDs upregulated the expression levels of *Atg6*, *Atg8*, and *Dronc* genes, although the time and amplitude of their upregulation after exposure varied, and gender-dependent differences were also found (Figs 7 and 8).

These results indicate that the mechanisms that underlie the toxicity of CdTe QDs in animal gonadal cells could be variable and complex, including apoptosis and autophagy via ROS-induced mitochondrial and lysosomal pathways, which occur during the *in vitro* cultivation of liver cells, lung cells, and cancer cells. After exposure to QDs, we found that the generation of ROS in the gonads as well as staining changes in the mitochondria and lysosomes demonstrated that QDs passed through the outer membranes of the gonads, where they first induced changes in the inner membrane cells and then spread to the germ cells developing inside the gonads.

## Conclusion

In this study, we found that dorsal vein injection of CdTe QDs induced the generation of ROS in the gonadal inner membranes and germ cells, which then affected reproduction via complex mechanisms associated with apoptosis and autophagy through mitochondrial and lysosomal pathways.

Exposure to QDs during the early stage of the life cycle had severe adverse effects on the quantity and quality of male sperm formation, which was the main cause of unfertilized eggs. Ala- or Gly-conjugated QDs can reduce the toxic effects of CdTe QDs during gonadal development and offspring fertilization.

## Methods

### Synthesis of CdTe QDs nanoparticles

Water-soluble CdTe QDs with maximum luminescent wavelengths of 530 nm (QDs) and two types of negative QDs with coating into amino acids, i.e., Ala (QDs-Ala) or Gly (QDs-Gly), were used in these experiments, which were synthesized based on our previously reported protocol^57^,^58^. Their corresponding hydrodynamic diameters were 3.6 nm, while their absorption and photoluminescence spectra, and their representative dynamic light scattering histograms were described in our previous report^16^. We found that the absorbances at 1600–1680, 2800–3000, and 3400 cm^−1^ were increased after the QDs were decorated with Ala or Gly. The absorbances at 1600–1680, 2800–3000, and 3400 cm^−1^, which were assigned to C=O bonds (stretching vibration), O-H bonds (stretching vibration), and N-H bonds (stretching vibration), respectively, indicated the presence of abundant carboxylic acid groups and amino bonds in the prepared QDs530-amino acid (Ala or Gly).

### Preparation of the test organism

*B. mori* strain Haoyue was reared on fresh mulberry leaves at 25 °C under a 12 h light and 12 h dark period regime with 70–90% relative humidity. At the beginning of the experiments, test organisms with similar sizes (1.74 ± 0.24 g) at the 48-h-old 5th instar were allocated randomly to test containers with a sufficient number of larvae in each, where one test group comprised three replicated containers. We administered 10 μL of the QDs by vascular injection, whereas the control organisms were injected with the same volume of pure water.

At an early stage, lethal toxicity studies were performed in order to assess the tolerance of the animals and to accurately determine the sub-lethal doses of QDs. We used 0.32 nmol (32 μM × 10 μL) and 0.64 nmol (64 μM × 10 μL) QDs, QDs-Ala, or QDs-Gly per individual to assess the reproductive toxicity. The larvae were reared until they became adult moths, which were used to assess chronic effects on reproduction in mating experiments. Furthermore, the testes and ovaries were removed carefully using surgical forceps at 6 h, 12 h, 24 h, 48 h, 72 h, 96 h, and 120 h after the injection of QDs, and employed to make morphological observations of gonads, for tissue culture, and other uses. The sex of the larvae could be distinguished before feeding in the newly molted 5th instar larvae based on their abdominal characteristics^59^ (Fig. S3).

All of the animal experimental procedures in this study were performed in accordance with Soochow University Guidelines for the Welfare of Animals.

### Reproduction testing

Using different mated pairs (n = 5), we investigated the effect of QDs on general fecundity based on the number of fertilized eggs laid per female, the oviposition performance of females according to the total eggs laid per female (include unfertilized eggs and fertilized eggs), the egg production capacity based on the total number of eggs per female (the total eggs laid plus the remaining eggs in ovarioles), and male fertility via the rate of fertilized eggs. The mated pairs were prepared according to the following procedures. 1) Un-exposed female adults were mated with un-exposed male adults. 2) Exposed female adults were mated with un-exposed male adults. 3) Un-exposed female adults were mated with exposed male adults. 4) Exposed female adults were mated with exposed male adults. The two sexes were easily distinguished based on their form and the abdominal characteristics of pupae or adults (Fig. S3).

The 5th instar larvae received vascular injection of 0.32 nmol CdTe QDs, QDs-Ala, or QDs-Gly per individual (10 μL at 32 μM) at 48 h after molting, whereas the control organisms were injected with 10 μL of pure water. The larvae were then reared on fresh mulberry leaves at 25 °C under a photoperiod of 12 h light and 12 h dark until they became adult moths. The adults were mated for 4 h with descriptive mates and the mated females were then transferred to separated iron hoops (cylindrical in shape: height = 4 cm, diameter = 5 cm) on craft paper where they laid their eggs within 24 h. Next, the ovaries were removed from moths to investigate the remaining eggs. The yellow unfertilized eggs and the puce fertilized eggs were measured at 72 h after oviposition.

### Fluorescent tracking of QDs

The testes and ovaries were carefully removed using surgical forceps at 12 h, 24 h, 48 h, and 72 h after QDs injection. The testes and ovaries could be distinguished based on their characteristic forms. The characteristic green fluorescence of CdTe QDs, QDs-Ala, and QDs-Gly in the testes and ovaries were observed at an excitation wavelength of 490 nm and emission wavelength of 530 nm using a fluorescence microscope (Olympus BX51, Tokyo, Japan), or with a CRI Maestro^TM^ system (Maestro™ 2 EX-RRO, Wisconsin, USA).

### Combined *in vivo/in vitro* investigation

Spermateleosis was affected by the duration of exposure to QDs *in vivo* and the duration of culture free from QDs *in vitro*. The 5th instar larvae received vascular injections of 0.32 nmol CdTe QDs, QDs-Ala, or QDs-Gly per larva (10 μL at 32 μM per individual) at 48 h after molting, whereas the control organisms were injected with the 10 μL of pure water. The testes were removed from male larva using surgical forceps at 12 h, 24 h, or 72 h after exposure to QDs, and they were washed with pre-cooled physiological saline and culture medium three times to eliminate adherent hemocytes.

Each testis was then thoroughly split with forceps to release spermatocysts into 500 μL of culture medium. Let sit for 5 min, then the spermatocysts that sank to the bottom in 100 μL medium were placed in a 24-well plate, which contained 400 μL of Grace’s insect cell culture medium supplemented with 10% silkworm hemolymph and an appropriate amount of antibiotics. Next, the exposed or unexposed spermatocysts were subjected to *in vitro* culture free from QDs at 25 °C and 50% of the medium was replaced every 72 h. At specific times, the morphology of the developing spermatocysts was observed with an inverted microscope (U-LH100L-3, Olympus, Japan) and we counted the numbers of 64, 128, and 256 primary spermatocytes in spermatocysts during spermiogenesis and spermatodesms using a blood cell counter (Thoma, Tokyo, Japan).

### Observations of organelle damage

To observe organelle damage, we employed the method described by Xing *et al*.^15^ Briefly, the mitochondria and lysosomes in cells were stained with 100 nM LysoTracker Red (C1046; Beyotime, Jiangsu, China) and 200 nM Mito-Tracker Green (C1048; Beyotime), respectively. Ten testes or ovaries were placed in 2 mL of staining liquid to analyze organelle damage. The samples were incubated for 20 min at 25 °C, before washing for 5 min with saline in the dark. Next, we used a fluorescence microscope (Olympus BX51) to observe green fluorescence in mitochondria and red fluorescence in lysosomes.

The nuclei of the damaged cells in sex glands were stained with diamidino-phenyl-indole (C1005, Beyotime, China). The testes or ovaries were removed carefully at 120 h after the injection of QDs and subjected to nuclear staining. The histiocytes were stained with DAPI after thoroughly splitting from the outer membrane of testes and ovaries with forceps. The cells were observed with a fluorescence microscope (Olympus BX51).

### Tissue staining

The reactive oxygen species (ROS) levels were measured using a ROS kit (S0033-1, Beyotime, China) according to the method described by Liu *et al*.^6^. Briefly, the gonads were collected in diethy pyrocarbonate (containing 0.7% NaCl) and after vortexing for 20 s in normal saline, they were rapidly placed into the ROS staining solution for 30 min and washed for 5 min with saline in the dark. Green fluorescence from the ROS was observed at an excitation wavelength of 488 nm and emission wavelength of 525 nm with a fluorescence microscope (Olympus BX51).

The testes or ovaries were removed at 24 h after the injection of QDs and subjected to HE staining after fixation and sectioning as described by Ji *et al*.^60^. The sections were stained with HE purchased from Invitrogen (Carlsbad, CA, USA).

### Gene expression analysis

Total RNA was isolated from the testes or ovaries at 6 h, 24 h, and 48 h after the injection of QDs using RNAiso Plus (TaKaRa, Dalian, China). The cDNA was synthesized using a PrimeScript™ RT (Perfect Real Time) Reagent Kit with gDNA Eraser (TaKaRa), according to the manufacturer’s instructions. Quantitative real-time reverse transcription–PCR (qRT–PCR) was used to analyze the mRNA transcript levels of *BmDronc*, *BmAtg6*, and *BmAtg8* genes. The *BmRP49* gene was used as an internal control. RT–PCR was performed using a total reaction volume of 20 μL with an ABI StepOnePlus™ Real-Time PCR System (Ambion, Foster City, CA, USA) and the fluorescent dye SYBR Premix Ex Taq (TaKaRa), according to the manufacturers’ instructions and the method described by Ji *et al*.^60^. The primers used in this study are listed in Supplementary Table S1.

### Data analyses

Statistical differences between the control and treated samples were detected using the parametric *t*-test with SPSS 17.0. All of the data were analyzed using a single factor one-way analysis of variance (ANOVA). *P* < 0.05 was considered to indicate significant differences.

## Additional Information

**How to cite this article**: Yan, S.-Q. *et al*. Reproductive toxicity and gender differences induced by cadmium telluride quantum dots in an invertebrate model organism. *Sci. Rep*. **6**, 34182; doi: 10.1038/srep34182 (2016).

## Supplementary Material

Supplementary Information
