# Supplementary material for: Reproductive toxicity and gender differences induced by cadmium telluride quantum dots in an invertebrate model organism
Source: Sci Rep. 2016 Sep 27;6:34182. doi: 10.1038/srep34182 (PMC5037452; doi:10.1038/srep34182)
Supplement: Supplementary Information [file srep34182-s1.doc]

Reproductive toxicity and gender differences induced by cadmium telluride quantum dots in an invertebrate model organism

Si-Qi Yan, Rui Xing, Yan-Feng Zhou, Yuan-Yuan Su, Kai-Le Li, JF Qiu, Yun-Hu Zhang, Ke-Qin Zhang, Yao He, Xiao-Pin Lu, Shi-Qing Xu


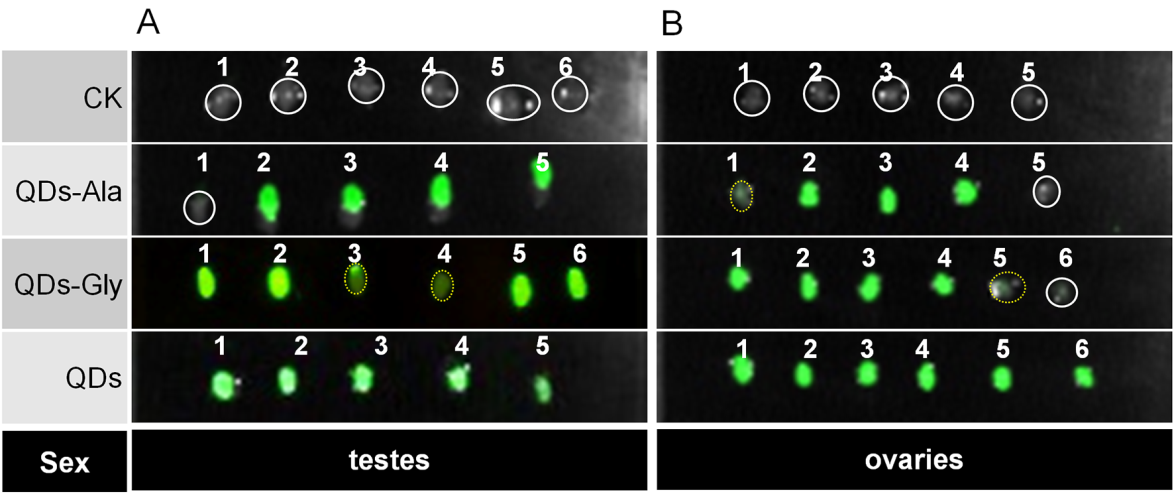


**Fig. S1 QDs were found in the** **testes (A) and ovaries (B) using the iBOX Scientia multispectral imaging system.** Merged images of white light and fluorescent light obtained by CRI Maestro^TM^ (Photometric, USA) showing the infiltration of QDs in gonads. Fifth instar larvae received vascular injection of 0.64 nmolCdTe QDs per larva (10 μL at 64 μM per individual) at 48 h after molting, whereas the control organisms (CK) were injected with the same volume of pure water. Gonads were removed from three male or female larvae at 12 h after exposure to QDs. The numbers in the figure show the amounts of intact testes or ovaries. The solid line boxes show non-fluorescent glands and the dashed line boxes show weakly fluorescent glands.


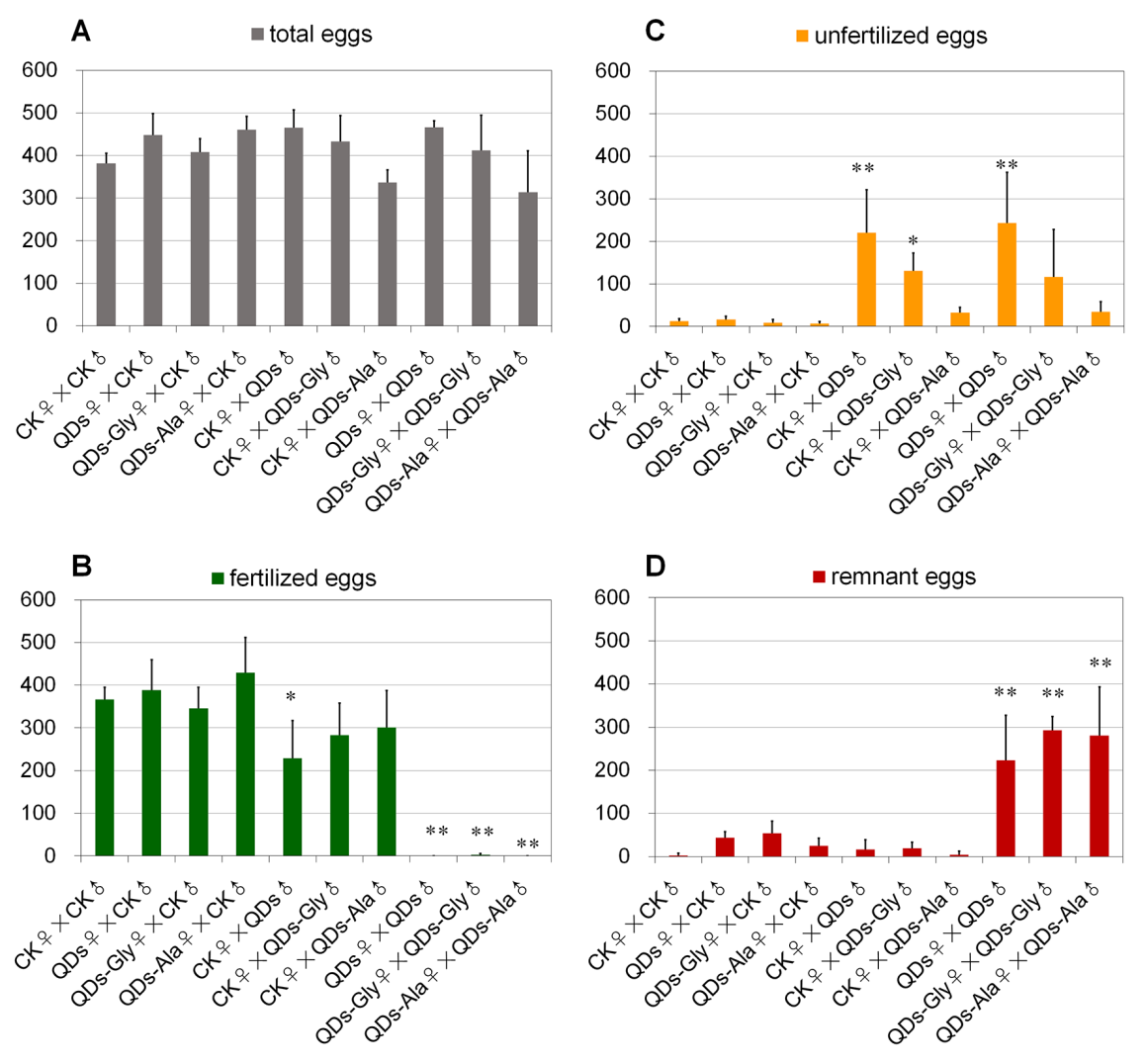


**Fig. S2 Effects of QDs on oviposition and fertilization. Numbers of total eggs (A), unfertilized eggs (B), fertilized eggs (C), and remaining eggs (D) in female moths.** Fifth instar larvae received vascular injection of 0.32 nmol CdTe QDs per individual (10 μL 32 μM) at 48 h after molting, whereas the control organisms (CK) were injected with the same volume of pure water. The larvae were reared on fresh mulberry leaves at 25 °C with a photoperiod of 12 h light and 12 h dark until they became adult moths. The adults were mated for 4 h with descriptive mates and the eggs were laid within 24 h. Next, the ovaries were removed from the moths to investigate the remaining eggs. The fertilized eggs were determined at 72 h after oviposition**.** n = 5 female moths. **P <* 0.05 and ***P <* 0.01 indicate significant differences compared with the control values.


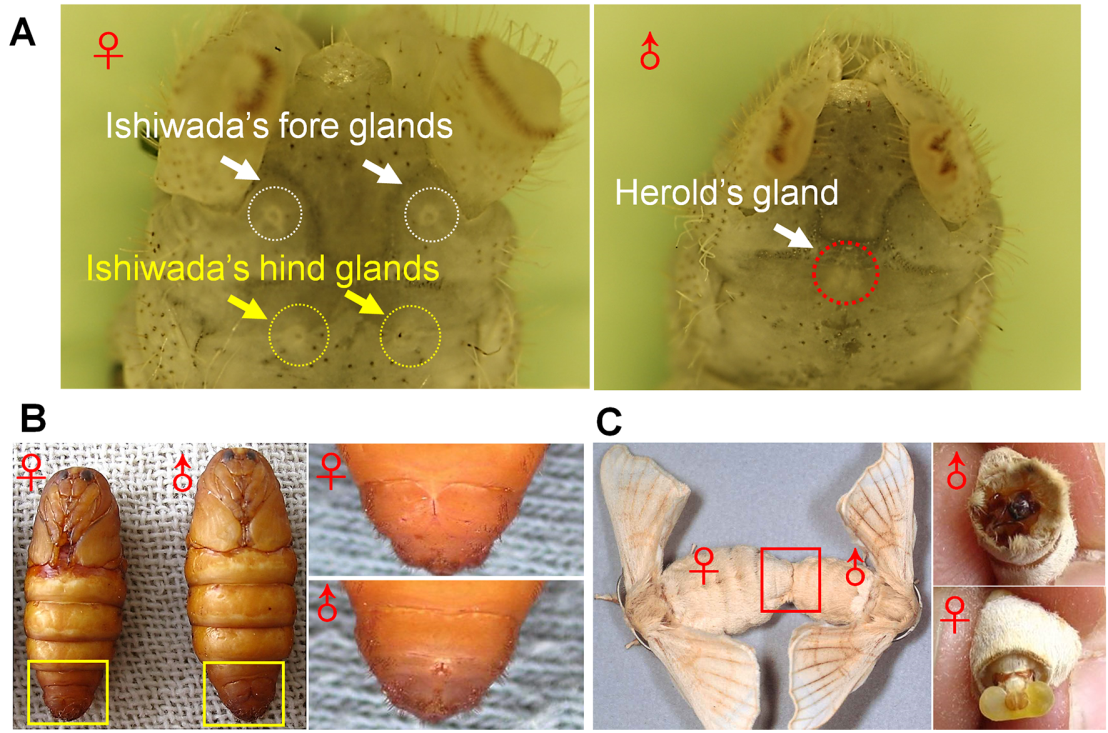


**Fig. S3. Sex distinction in *Bombyx mori* during the larval (A), pupal (B), and adult (C) stages.** The sexes of larvae could be distinguished before feeding in newly molted 5th instar larvae based on the four Ishiwada’s glands in females and Herold’s gland in males. The two sexes were easily distinguished based on their form and abdominal characteristics in pupae, and the caudal characteristics of adults.

Table S1 Primer sequences used for qRT–PCR

| Gene | Primer sequence |
| --- | --- |
| *BmAtg6* | S-GTTATACGGTTCGGGTGG |
|  | A-TGGAGTACGCATGTGGTG |
| *BmAtg8* | S-AAGGCTAGGCTTGGAGAC |
|  | A-CAGATGTGGGTGGAATGA |
| *BmDronc* | S-TGTGGCTGTCTTCCTTC |
|  | A-ATCTAAGTCTGTGCCCTC |
| *BmRp49* | S-CGATCCGCCGACGTTACTACA |
|  | A-GTCCGGGCCTGGTGAGATTT |
